# Insulin resistance and reduced cardiac autonomic function in older adults: the Atherosclerosis Risk in Communities study

**DOI:** 10.1186/s12872-020-01496-z

**Published:** 2020-05-11

**Authors:** Anna K. Poon, Eric A. Whitsel, Gerardo Heiss, Elsayed Z. Soliman, Lynne E. Wagenknecht, Takeki Suzuki, Laura Loehr

**Affiliations:** 1 Department of Epidemiology, University of North Carolina at Chapel Hill, Chapel Hill, NC USA; 2 Department of Medicine, University of North Carolina at Chapel Hill, Chapel Hill, NC USA; 3 Division of Epidemiology and Prevention, Wake Forest School of Medicine, Winston-Salem, NC USA; 4 Division of Public Health Sciences, Wake Forest School of Medicine, Winston-Salem, NC USA; 5 Department of Medicine, Indiana University School of Medicine, Indianapolis, IN USA

**Keywords:** Insulin resistance, Homeostatic model assessment of insulin resistance, Cardiac autonomic function, Ambulatory electrocardiograms, Heart rate variability

## Abstract

**Background:**

Prior studies have shown insulin resistance is associated with reduced cardiac autonomic function measured at rest, but few studies have determined whether insulin resistance is associated with reduced cardiac autonomic function measured during daily activities.

**Methods:**

We examined older adults without diabetes with 48-h ambulatory electrocardiography (*n* = 759) in an ancillary study of the Atherosclerosis Risk in Communities Study. Insulin resistance, the exposure, was defined by quartiles for three indexes: 1) the homeostatic model assessment of insulin resistance (HOMA-IR), 2) the triglyceride and glucose index (TyG), and 3) the triglyceride to high-density lipoprotein cholesterol ratio (TG/HDL-C). Low heart rate variability, the outcome, was defined by <25th percentile for four measures: 1) standard deviation of normal-to-normal R-R intervals (SDNN), a measure of total variability; 2) root mean square of successive differences in normal-to-normal R-R intervals (RMSSD), a measure of vagal activity; 3) low frequency spectral component (LF), a measure of sympathetic and vagal activity; and 4) high frequency spectral component (HF), a measure of vagal activity. Logistic regression was used to estimate odds ratios (OR) and 95% confidence intervals weighted for sampling/non-response, adjusted for age at ancillary visit, sex, and race/study-site. Insulin resistance quartiles 4, 3, and 2 were compared to quartile 1; high indexes refer to quartile 4 versus quartile 1.

**Results:**

The average age was 78 years, 66% (*n* = 497) were women, and 58% (*n* = 438) were African American. Estimates of association were not robust at all levels of HOMA-IR, TyG, and TG/HDL-C, but suggest that high indexes were associated consistently with indicators of vagal activity. High HOMA-IR, high TyG, and high TG/HDL-C were consistently associated with low RMSSD (OR: 1.68 (1.00, 2.81), OR: 2.03 (1.21, 3.39), and OR: 1.73 (1.01, 2.91), respectively). High HOMA-IR, high TyG, and high TG/HDL-C were consistently associated with low HF (OR: 1.90 (1.14, 3.18), OR: 1.98 (1.21, 3.25), and OR: 1.76 (1.07, 2.90), respectively).

**Conclusions:**

In older adults without diabetes, insulin resistance was associated with reduced cardiac autonomic function – specifically and consistently for indicators of vagal activity – measured during daily activities. Primary prevention of insulin resistance may reduce the related risk of cardiac autonomic dysfunction.

## Background

Insulin resistance is a condition defined by a reduced response to insulin in target tissues [1]. It often precedes the development of the metabolic dysregulations and metabolic disorders that contribute to an increase in risk for cardiovascular disease [2–5]. However, insulin resistance is modifiable [6], making it an important target for primary prevention of cardiovascular risk.

Insulin resistance may contribute to cardiovascular risk attributed, in part, to its effect on cardiac autonomic function [7] that can be measured non-invasively with heart rate variability [8] and has been shown to be associated with a higher incidence of cardiovascular events [9]. Prior studies have shown that adults with diabetes have lower heart rate variability [10–12]. Furthermore, among adults without diabetes, heart rate variability is lower in adults with elevated insulin levels [10–12]. This evidence is based on shorter-term electrocardiogram recordings measured at rest. There is a limited understanding based on longer-term electrocardiogram recordings measured during daily activities.

Insulin resistance can be measured using insulin resistance indexes that are less invasive than standard reference measures [13] and represent other aspects of insulin resistance, such as hepatic insulin resistance and peripheral insulin resistance. They include the homeostatic model assessment of insulin resistance (HOMA-IR) [14], the triglyceride and glucose index (TyG) [15], and the triglyceride to high-density lipoprotein cholesterol ratio (TG/HDL-C) [16]. The use of more than one insulin resistance index can reflect the effect of different aspects of insulin resistance in exposure-outcome relationships.

An understanding of the relationship between insulin resistance indexes and cardiac autonomic function may improve our understanding of the contribution to cardiovascular risk attributed to cardiac autonomic function. Our goal was to determine the association of insulin resistance indexes with cardiac autonomic function – measured by 48-h ambulatory electrocardiogram monitoring – in a population of older adults without diabetes.

## Methods

### Study population

This study [17] was ancillary to the Atherosclerosis Risk in Communities (ARIC) Study [18], an ongoing prospective study of the causes of atherosclerosis and its clinical outcomes in adults 45 to 64 years of age at recruitment in 1987–1989. This study invited participants from among those who attended the ARIC Study Visit 5 cohort exam to attend a brief clinic visit that included placement of a 48-h ambulatory electrocardiogram monitor; the age range at enrollment was 66 to 89. For this analysis, the exposure and covariates, unless otherwise noted, were measured at the Visit 5 cohort exam (2011–2013) and the outcome measure of heart rate variability was measured at the ancillary study visit (2014–2016). Participants were selected using a stratified random sampling design to enrich for African-Americans and risk factors for atrial fibrillation. Participants were selected from 2 (Forsyth County, North Carolina and Jackson, Mississippi) of the 4 ARIC field centers; and considered at high risk of atrial fibrillation if they had a prior hospitalization for heart failure, reduced ejection fraction (< 50%), or enlarged left atrial size (left atrial volume index ≥ 32) on echocardiography at Visit 5. A total of 1205 participants were included in the study. This study was approved by the Institutional Review Boards at the field centers and the Collaborative Studies Coordinating Center at the University of North Carolina at Chapel Hill. All participants provided written informed consent at the visits.

For our analysis, we excluded adults with missing ambulatory electrocardiograms due to transmission issues or dropout (*n* = 3 and *n* = 1, respectively); ambulatory electrocardiograms with > 10% noise or recording time < 20 h (*n* = 4); missing medication information at either visit (n = 4); use of antiarrhythmic medications at either visit (*n* = 10); diabetes at Visit 5 or insulin medications at Visit 5 or insulin medications at the ancillary visit (*n* = 383); and fasting < 8 h at Visit 5 (*n* = 41). Insulin resistance indexes ± 3SD from the mean were excluded. After exclusions, our analytic sample included *n* = 759 adults without diabetes. After distribution-based exclusions, our final analytic sample included *n* = 727 adults with HOMA-IR values, *n* = 749 adults with TyG values, and *n* = 750 adults with TG/HDL-C values. See Supplemental Figure 1 for a flow chart of the exclusions.

### Blood collection, processing, and assay

Blood specimens were collected using a standardized venipuncture protocol, processed within 90 min, and shipped weekly to central laboratories at Visit 5 [19]. Fasting glucose was assayed using enzymatic methods. Fasting insulin was assayed using immunoassay methods. Triglyceride was assayed using enzymatic methods. High-density lipoprotein cholesterol was assayed using direct methods. The coefficient of variation was 10.6% (mean 12.9 μU/mL) for fasting insulin, 3.1% (mean 112.9 mg/dL) for fasting glucose, 4.9% (mean 125.2 mg/dL) for triglyceride, and 4.2% (mean 51.7 mg/dL) for high-density lipoprotein cholesterol [20].

### Insulin resistance indexes

Insulin resistance indexes were calculated using measures from Visit 5. The homeostatic model assessment of insulin resistance (HOMA-IR) was equal to: (fasting glucose in mg/dL x fasting insulin in μU/mL) / 405. The triglyceride and glucose index (TyG) was equal to: Ln [(fasting triglyceride in mg/dL x fasting glucose in mg/dL) / 2]. The triglyceride to high-density lipoprotein cholesterol ratio (TG/HDL-C) was equal to: (fasting triglyceride in mg/dL / fasting high-density lipoprotein cholesterol in mg/dL). The repeatability of HOMA-IR and TG/HDL-C -- measured as intraclass correlation coefficients among participants examined twice, 4–15 weeks apart -- was 0.70 for HOMA-IR and 0.80 for TG/HDL-C [20]. The repeatability of TyG was not available, but the repeatability of its constituent analytes -- triglyceride and fasting glucose -- was available; the short-term repeatability was 0.76 for triglyceride and 0.56 for fasting glucose [20].

### Ambulatory electrocardiograms

An ambulatory electrocardiogram monitor (SEER Light Extend; GE, Milwaukee, WI) was attached using 7 electrodes in a modified V3 placement using a standardized protocol at the brief ancillary visit. Participants were instructed to wear the monitor during usual activities, but to avoid getting it wet and to return it after 48 h. Electrocardiographic recordings were digitally transferred to, and centrally processed by, the Epidemiological Cardiology Research Center at the Wake Forest School of Medicine. Recordings were analyzed by trained technicians following a standardized protocol (GE MARS 8.0.2; GE, Milwaukee, Wisconsin).

### Heart rate variability

Heart rate variability metrics were derived using 48-h ambulatory electrocardiography from the ancillary study visit. Heart rate variability was based on an analysis of the intervals between successive normal QRS complexes. Time domain measures included the standard deviation of normal-to-normal R-R intervals (SDNN), a measure of total variability; the root mean square of successive differences in normal-to-normal R-R intervals (RMSSD), a measure of vagal activity; and the percentage of successive normal-to-normal R-R intervals that differ by > 50 ms (pNN50), a measure of vagal activity. Frequency domain measures included the low-frequency spectral component (LF) from 0.04 to 0.15 Hz, a measure of sympathetic and vagal activity; the high-frequency spectral component (HF) from 0.15 to 0.4 Hz, a measure of vagal activity; and the ratio of low frequency to high frequency (LF/HF), a measure of sympathovagal balance.

### Covariates

Participant characteristics were measured using a standardized protocol at Visit 5 [21]. Blood pressure was measured using a sphygmomanometer after a five-minute waiting period; three measurements were taken and the mean of the last two of three measurements was used in analyses. Waist circumference was measured using a measuring tape, at the apex of the iliac crest and at the end of a normal expiration. Weight was measured using a digital scale, height was measured using a fixed stadiometer, and body mass index was calculated as weight in kilograms divided by height in meters squared. Former smoker (yes vs no) status and former drinker (yes vs no) status were ascertained via self-report on questionnaires. Any prior coronary heart disease was based on self-report at Visit 1 in 1987–1989, ongoing surveillance of hospitalizations in the communities, and hospitalizations that occurred outside the communities as identified from annual follow-up of participants [18].

### Statistical analysis

Participant characteristics were described overall and by quartiles of insulin resistance indexes. Associations between the insulin resistance index quartiles and participant characteristics were assessed using ANOVA for continuous variables and the chi-squared test for categorical variables.

Logistic regression with weights for sampling and non-response was used to estimate odds ratios (OR) and 95% confidence intervals (95% CI). Insulin resistance indexes were defined by quartiles. Low heart rate variability was defined by metrics <25th percentile. The estimates of association for quartiles 4, 3, and 2 (i.e., the comparison groups) were compared to quartile 1 (i.e., the referent group); and the Cochran-Armitage test was used to test for trend. The estimates are interpreted as the prospective association of insulin resistance indexes with reduced cardiac autonomic function.

In the main analysis, age at ancillary visit, sex, and race/study-site (Jackson site/African-American, Forsyth site/White, Forsyth site/African-American) were used as covariates because they were considered potential confounders. In supplemental analysis, any prior coronary heart disease and systolic blood pressure were used as covariates because they were considered potential confounders. Additional analysis was conducted to examine the estimates by race.

Insulin resistance indexes were defined using quartiles (non-linear terms) and standardized units (linear terms), then the Akaike information criterion was used to select for fit of functional form. Because the Akaike information criterion indicated the fit was similar for both non-linear terms and linear terms, non-linear terms were used for the estimates of association.

We observed an association between insulin resistance indexes and heart rate variability metrics that were indicative of vagal activity. We decided to further investigate the relationship between insulin resistance indexes and vagal activity by including pNN50; and the relationship between insulin resistance indexes and sympathovagal balance by including the LF/HF ratio. We defined pNN50 as <25th percentile and LF/HF as >75th percentile.

## Results

The average age was 78 years, 66% (*n* = 497) were women, and, per the sampling design, 58% (*n* = 438) were African American. Waist circumference was higher with higher quartiles of HOMA-IR; the trend was similar by quartiles of TyG and by quartiles of TG/HDL-C. Systolic blood pressure was not higher but use of blood pressure medications was higher with higher quartiles of HOMA-IR (Table 1); however, the relationship differed by quartiles of TyG (Supplemental Table 2, Additional file 1) and TG/HDL-C (Supplemental Table 3, Additional file 1).

**Table 1:** Participant characteristics by quartiles of homeostatic model assessment of insulin resistance (HOMA-IR) (Visit 5, 2011–2013)

	HOMA-IR					
	Quartile 1	Quartile 2	Quartile 3	Quartile 4	All	*P*-value
	[0.20, 1.73]	[1.74, 2.78]	[2.79, 4.34]	[4.35,16.88]		
	(*n* = 181)	(*n* = 182)	(*n* = 182)	(n = 182)	(n = 727)	
Age (years), mean ± SE	78 ± 0.4	78 ± 0.4	77 ± 0.3	77 ± 0.3	78 ± 0.2	< 0.01
Women, n(%)	124 (69)	123 (68)	109 (60)	116 (64)	497 (66)	0.17
African American, n(%)	100 (55)	105 (58)	102 (56)	115 (63)	438 (58)	0.18
Waist circumference (cm), mean ± SE	89 ± 0.7	95 ± 0.9	100 ± 0.8	106 ± 1	97 ± 0.5	< 0.0001
BMI (kg/m^2^), mean ± SE	25 ± 0.3	28 ± 0.4	29 ± 0.4	32 ± 0.5	29 ± 0.2	< 0.0001
SBP (mmHg), mean ± SE	129 ± 1.4	129 ± 1.5	128 ± 1.2	128 ± 1.3	129 ± 0.7	0.91
DBP (mmHg), mean ± SE	64 ± 0.9	65 ± 0.8	64 ± 0.7	65 ± 0.8	65 ± 0.4	0.78
Heart rate (beats per minute), mean ± SE	72 ± 0.6	72 ± 0.7	72 ± 0.6	71 ± 0.7	72 ± 0.3	0.79
Blood pressure medication, n(%)	104 (58)	120 (67)	123 (69)	137 (76)	510 (68)	0.02
Prior CHD, n(%)	16 (9)	25 (14)	20 (11)	27 (15)	91 (12)	0.16
Current smoker, n(%)	14 (8)	7 (4)	12 (7)	6 (3)	39 (5)	0.13
Current drinker, n(%)	60 (36)	60 (35)	56 (32)	54 (30)	238 (33)	0.22
Former smoker, n(%)	56 (36)	84 (54)	87 (54)	82 (50)	325 (49)	0.02
Former drinker, n(%)	48 (28)	62 (36)	65 (37)	67 (37)	258 (36)	0.09
Fasting glucose (mg/dL), mean ± SE	95 ± 0.7	102 ± 0.7	105 ± 0.6	108 ± 0.7	103 ± 0.4	< 0.0001
Insulin (μU/mL), mean ± SE	5 ± 0.1	9 ± 0.1	13 ± 0.2	24 ± 0.6	13 ± 0.3	< 0.0001

Participant characteristics were described overall and by quartiles of insulin resistance indexes. Continuous variables were described using ANOVA and categorical variables were described using the chi-squared test

**Fig. 1:**
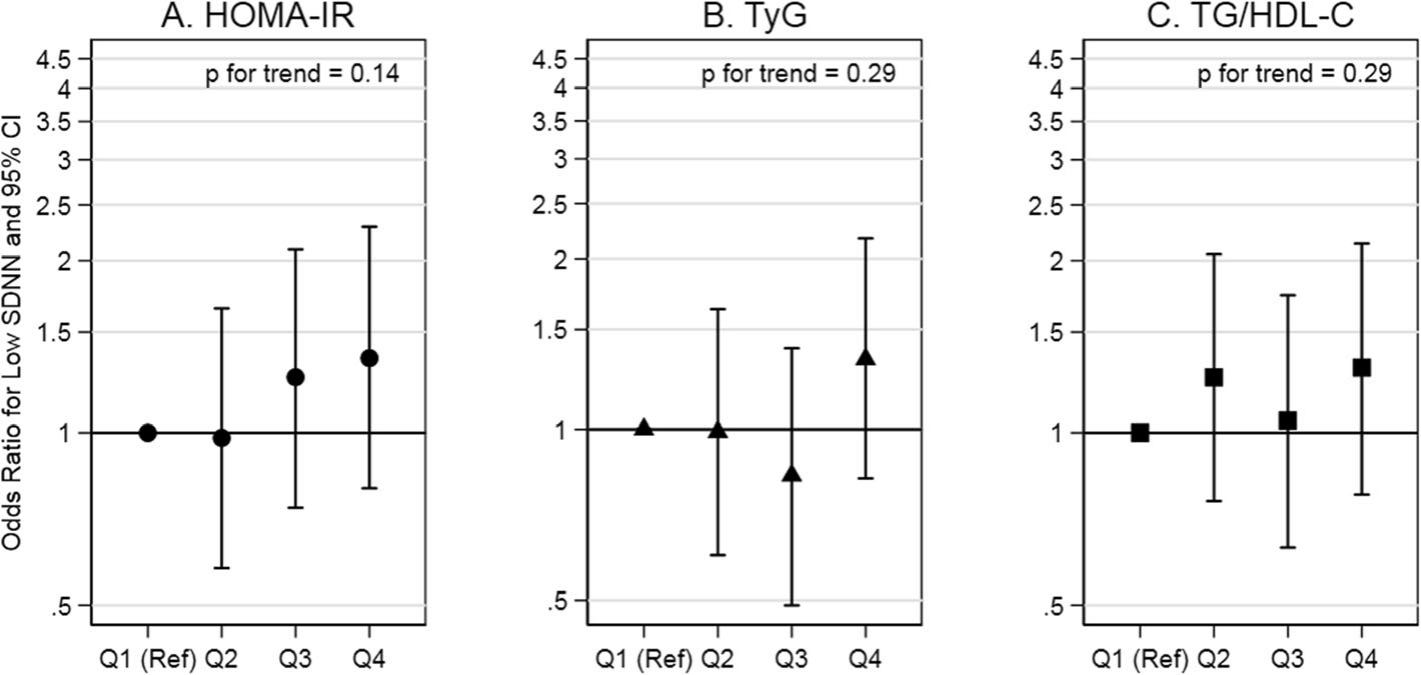
Association of insulin resistance indexes with low SDNN in adults without diabetes. Abbreviations: HOMA-IR, homeostatic model assessment of insulin resistance; TyG, triglyceride and glucose index; TG/HDL-C, triglyceride to high-density lipoprotein cholesterol ratio; SDNN, standard deviation of normal-to-normal R-R intervals. Comments: HOMA-IR quartiles are equal to: [0.20, 1.73], [1.74, 2.78], [2.79, 4.34], and [4.35, 16.88]. TyG quartiles are equal to: [7.25, 8.24], [8.25, 8.54], [8.55, 8.81], and [8.82, 9.93]. TG/HDL-C quartiles are equal to: [0.45, 1.29], [1.30, 1.95], [1.96, 2.74], and [2.75, 9.30]. Low SDNN (<25th percentile) is equal to < 97 ms. Estimates are adjusted for age at ancillary visit, sex, and race/study-site

**Fig. 2:**
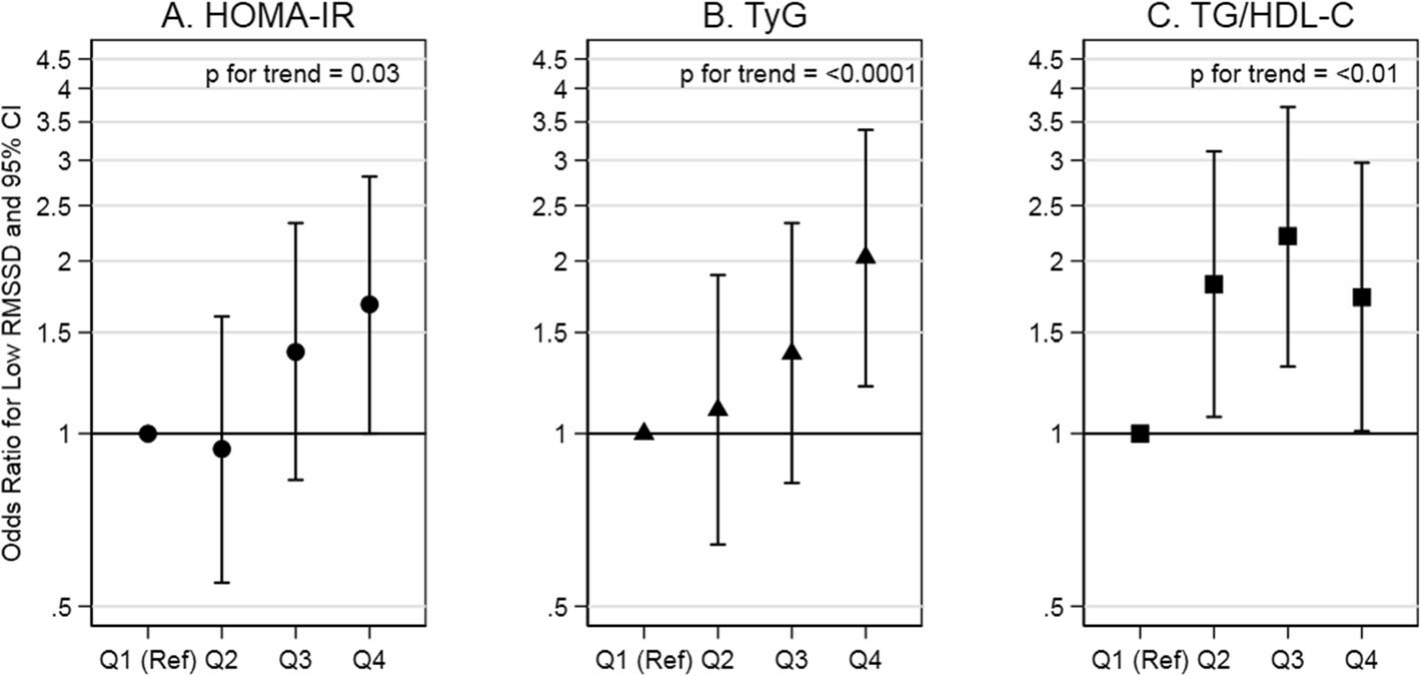
Association of insulin resistance indexes with low RMSSD in adults without diabetes. Abbreviations: HOMA-IR, homeostatic model assessment of insulin resistance; TyG, triglyceride and glucose index; TG/HDL-C, triglyceride to high-density lipoprotein cholesterol ratio; RMSSD, root mean square of successive differences in normal-to-normal R-R intervals. Comments: HOMA-IR quartiles are equal to: [0.20, 1.73], [1.74, 2.78], [2.79, 4.34], and [4.35, 16.88]. TyG quartiles are equal to: [7.25, 8.24], [8.25, 8.54], [8.55, 8.81], and [8.82, 9.93]. TG/HDL-C quartiles are equal to: [0.45, 1.29], [1.30, 1.95], [1.96, 2.74], and [2.75, 9.30]. Low RMSSD (<25th percentile) is equal to < 21 ms. Estimates are adjusted for age at ancillary visit, sex, and race/study-site

High insulin resistance indexes (i.e., Quartile 4 vs Quartile 1) were not associated with low SDNN, but were associated with low RMSSD. Low SDNN was as likely at low insulin resistance indexes as at high insulin resistance indexes for HOMA-IR, TyG, and TG/HDL-C (OR: 1.35 (0.80, 2.29), OR: 1.33 (0.82, 2.17), OR: 1.30 (0.78, 2.14)). The test for trend indicated no association between any of the indexes and low SDNN. Low RMSSD was more likely at high, as compared to low, insulin resistance indexes for HOMA-IR, TyG, and TG/HDL-C (OR: 1.68 (1.00, 2.81), OR: 2.03 (1.21, 3.39), and OR: 1.73 (1.01, 2.97), respectively). The test for trend indicated a positive association between the indexes and low RMSSD. In summary, there was a lack of an association of insulin resistance indexes with low SDNN, an indicator of total heart rate variability (Fig. 1; Supplemental Table 1, Additional file 1), but a positive association with low RMSSD, an indicator of vagal activity (Fig. 2; Supplemental Table 1, Additional file 1).

**Fig. 3:**
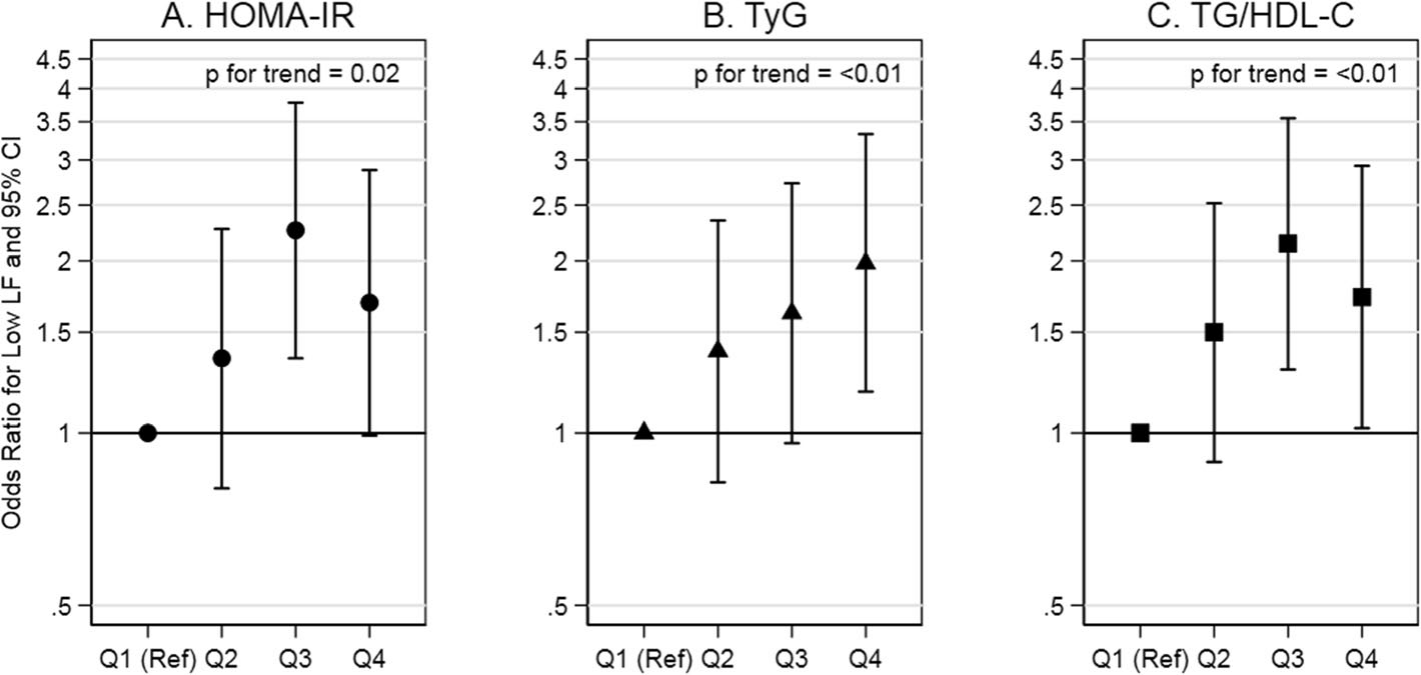
Association of insulin resistance indexes with low LF in adults without diabetes. Abbreviations: HOMA-IR, homeostatic model assessment of insulin resistance; TyG, triglyceride and glucose index; TG/HDL-C, triglyceride to high-density lipoprotein cholesterol ratio; LF, low frequency. Comments: HOMA-IR quartiles are equal to: [0.20, 1.73], [1.74, 2.78], [2.79, 4.34], and [4.35, 16.88]. TyG quartiles are equal to: [7.25, 8.24], [8.25, 8.54], [8.55, 8.81], and [8.82, 9.93]. TG/HDL-C quartiles are equal to: [0.45, 1.29], [1.30, 1.95], [1.96, 2.74], and [2.75, 9.30]. Low LF (<25th percentile) is equal to < 7.59 Hz. Estimates are adjusted for age at ancillary visit, sex, and race/study-site

**Fig. 4:**
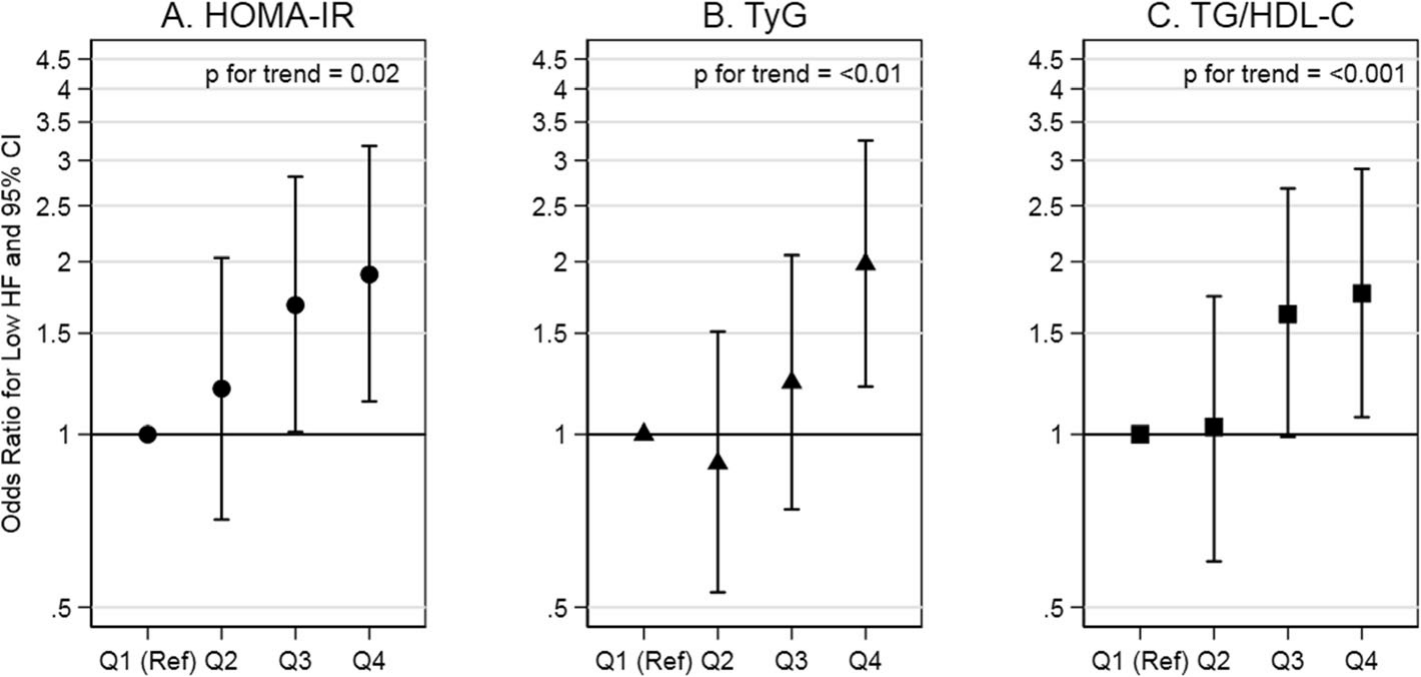
Association of insulin resistance indexes with low HF in adults without diabetes. Abbreviations: HOMA-IR, homeostatic model assessment of insulin resistance; TyG, triglyceride and glucose index; TG/HDL-C, triglyceride to high-density lipoprotein cholesterol ratio; HF, high frequency. Comments: HOMA-IR quartiles are equal to: [0.20, 1.73], [1.74, 2.78], [2.79, 4.34], and [4.35, 16.88]. TyG quartiles are equal to: [7.25, 8.24], [8.25, 8.54], [8.55, 8.81], and [8.82, 9.93]. TG/HDL-C quartiles are equal to: [0.45, 1.29], [1.30, 1.95], [1.96, 2.74], and [2.75, 9.30]. Low HF (<25th percentile) is equal to < 10.97 Hz. Estimates are adjusted for age at ancillary visit, sex, and race/study-site

High insulin resistance indexes (i.e., Quartile 4 vs Quartile 1) were associated with low LF and low HF. Compared to low insulin resistance indexes, low LF was more likely at high insulin resistance indexes for HOMA-IR although not statistically significant, for TyG, and for TG/HDL-C (OR: 1.69 (0.99, 2.88), OR: 1.98 (1.18, 3.33), and OR: 1.73 (1.02, 2.93), respectively). The test for trend indicated a positive association between the indexes and low LF. Compared to low insulin resistance indexes, low HF was more likely at high insulin resistance indexes for HOMA-IR, TyG, and TG/HDL-C (OR: 1.90 (1.14, 3.18), OR: 1.98 (1.21, 3.25), and OR: 1.76 (1.07, 2.90), respectively). The test for trend indicated a positive association between the indexes and low HF. In summary, there was a positive association of insulin resistance indexes with low LF, an indicator of sympathetic and vagal activity (Fig. 3; Supplemental Table 1, Additional file 1), and with low HF, an indicator of vagal activity (Fig. 4; Supplemental Table 1, Additional file 1).

Adjusting for any prior coronary heart disease and systolic blood pressure did not substantively alter the estimates. The inferences remained the same (Supplemental Tables 4 and 5, respectively, Additional file 1). Stratifying by race did not indicate heterogeneity by race. The inferences remained the same (Supplemental Tables 6 and 7, Additional file 1). We did not observe an association between the insulin resistance indexes with pNN50 or with LF/HF (Supplemental Table 8, Additional file 1).

## Discussion

We examined the relationship between insulin resistance and cardiac autonomic function measured during daily activities using ambulatory electrocardiography recordings in older adults in a large population-based cohort study. We observed the estimates were not robust for all measures of insulin resistance with all measures of cardiac autonomic function, but were robust with measures of cardiac autonomic function indicative of vagal activity. As such, higher insulin resistance indexes were associated with reduced cardiac autonomic function -- consistently and specifically -- for indicators of vagal activity.

Prior studies reported on the relationship between insulin resistance that used fasting insulin and heart rate variability. A prior study of middle age adults without diabetes showed an inverse relationship between insulin and SDNN, as well as between insulin and RMSSD using 2-min electrocardiography recordings (*n* = 8971) [11]. A similar study showed an inverse relationship between insulin and HF using 2-min electrocardiography recordings (*n* = 1779) [12]. Finally, a previous study of adults without diabetes showed an inverse association of log insulin (and log-HOMA-IR) with SDNN, RMSSD, log LF, and log HF using 24-h electrocardiography recordings (*n* = 94) [22]. Whereas prior studies defined cardiac autonomic dysfunction as the exposure, our study instead defined insulin resistance as the exposure (i.e., insulin resistance as an antecedent to cardiac autonomic dysfunction) [23]. Compared to studies with electrocardiography recordings measured at rest, we report similar inferences using ambulatory recordings measured during daily activities. Our findings suggest that the effect, reduced regulation (variation) of heart rate, is sustained, on average, over time.

We investigated the role of other metrics of heart rate variability. We did not observe a relationship between the insulin resistance indexes with vagal activity defined by pNN50. This result may be because the meaning of this metric may depend on the attributes of the study population and length of monitoring [24]; as such, we observed a relationship with some but not all metrics of vagal activity. We did not observe a relationship between the insulin resistance indexes with sympathovagal imbalance defined by LF/HF. This result may be because this metric can depend on the make-up of its component metrics [25]. Because the relationship between sympathetic activity and parasympathetic activity is non-reciprocal (i.e., not mutual), this metric may not always be an accurate representation of sympathovagal balance.

Insulin has been shown to regulate sympathetic and parasympathetic activity [26–31]. In insulin sensitive adults, the response to insulin is a shift from sympathetic activity to parasympathetic activity, whereas in insulin resistant adults, the response is limited (i.e., less in increment) [29]. This effect may be attributed to reduced response of the sinoatrial node to sympathetic and parasympathetic activity [30]. Insulin resistance may thus contribute to cardiac autonomic dysfunction, in the absence of diabetes.

Our study should be considered in the context of its strengths and limitations. We assessed insulin resistance with insulin resistance indexes instead of standard reference measures such as the euglycemic hyperinsulinemic clamp. Although there is a link in the dysregulation of glucose, insulin, and lipids in insulin resistance, the role of lipids may be affected by or influenced by pathways independent of insulin resistance [32]. However, prior studies have indicated a strong correlation of HOMA-IR (r = − 0.820) [14], TyG (r = − 0.681) [15], and TG/HDL-C (r = 0.60) [16] with direct measures of insulin mediated glucose uptake. We observed that the statistical significance of the estimates varied indicating that the estimates of association may not be robust with respect to HOMA-IR, TyG, and TG/HDL-C. However, although the statistical significance of the estimates may be dissimilar, the magnitude and direction of the estimates are similar for HOMA-IR, TyG, and TG/HDL-C, suggesting a consistent relationship in our study. Our strengths include the use of a cohort that is well-characterized and a focus on adults without diabetes.

The relationship between insulin resistance and cardiac autonomic function is important for public health; insulin resistance can be modified through lifestyle changes. Insulin resistance often precedes the development of diabetes [33] that has been shown to be associated with cardiac dysinnervation [34]. Insulin resistance itself may contribute to cardiac autonomic dysfunction. Because insulin resistance can be improved with lifestyle-based interventions, it is an important target for primary prevention [6].

## Conclusions

In summary, higher levels of insulin resistance indexes were associated with reduced cardiac autonomic function -- consistently and specifically for indicators of vagal activity – measured during daily activities in older adults without diabetes, using ambulatory electrocardiographic monitoring in a large population-based study. Primary prevention of insulin resistance may reduce the risk of cardiac autonomic dysfunction.

## Supplementary information

**Additional file 1: Table S1.** Odds ratios and 95% confidence intervals for main analysis. **Table S2.** Participant characteristics by quartiles of triglyceride and glucose index (TyG) (Visit 5, 2011–2013). **Table S3.** Participant characteristics by quartiles of triglyceride to high-density lipoprotein cholesterol ratio (TG/HDL-C) (Visit 5, 2011–2013). **Table S4.** Odds ratios and 95% confidence intervals for additional analysis, adjusting for any prior coronary heart disease. **Table S5.** Odds ratios and 95% confidence intervals for additional analysis, adjusting for systolic blood pressure. **Table S6.** Odds ratios and 95% confidence intervals for African American participants. **Table S7.** Odds ratios and 95% confidence intervals for White participants. **Table S8.** Odds ratios and 95% confidence intervals for additional analysis, including other outcome metrics. **Figure S1.** Study Population.

## Data Availability

The data to support the findings of this study are available from the Atherosclerosis Risk in Communities Study. The data are available upon request from the authors and permission from the Atherosclerosis Risk in Communities Study.

## Abbreviations

HOMA-IR: Homeostatic model assessment of insulin resistance
TyG: Triglyceride and glucose index
TG/HDL-C: Triglyceride to high-density lipoprotein cholesterol ratio
SDNN: Standard deviation of normal-to-normal R-R intervals
RMSSD: Root mean square of successive differences in normal-to-normal R-R intervals
LF: Low frequency spectral component
HF: High frequency spectral component
