# Supplementary material for: Insulin resistance and reduced cardiac autonomic function in older adults: the Atherosclerosis Risk in Communities study
Source: BMC Cardiovasc Disord. 2020 May 11;20:217. doi: 10.1186/s12872-020-01496-z (PMC7216367; doi:10.1186/s12872-020-01496-z)

**Supplemental Table 1.** Odds ratios and 95% confidence intervals for main analysis

|  | Low SDNN |  | Low RMSSD |  | Low LF |  | Low HF |  |
| --- | --- | --- | --- | --- | --- | --- | --- | --- |
|  | (<97 ms) |  | (<21 ms) |  | (<10.97 Hz) |  | (<7.59 Hz) |  |
|  | OR (95% CI) | P-value | OR (95% CI) | P-value | OR (95% CI) | P-value | OR (95% CI) | P-value |
| HOMA-IR |  | 0.14 |  | **0.03** |  | **0.02** |  | **0.02** |
| 4th Quartile [4.35,16.88] | 1.35 (0.80, 2.29) |  | **1.68 (1.00, 2.81)** |  | 1.69 (0.99, 2.88) |  | **1.90 (1.14, 3.18)** |  |
| 3rd Quartile [2.79, 4.34] | 1.25 (0.74, 2.09) |  | 1.39 (0.83, 2.33) |  | **2.26 (1.35, 3.78)** |  | **1.68 (1.01, 2.81)** |  |
| 2nd Quartile [1.74, 2.78] | 0.98 (0.58, 1.65) |  | 0.94 (0.55, 1.60) |  | 1.35 (0.80, 2.27) |  | 1.20 (0.71, 2.03) |  |
| 1st Quartile [0.20, 1.73] | REF |  | REF |  | REF |  | REF |  |
|  |  |  |  |  |  |  |  |  |
| TyG |  | 0.29 |  | **<0.0001** |  | **<0.01** |  | **<0.01** |
| 4th Quartile [8.82, 9.93] | 1.33 (0.82, 2.17) |  | **2.03 (1.21, 3.39)** |  | **1.98 (1.18, 3.33)** |  | **1.98 (1.21, 3.25)** |  |
| 3rd Quartile [8.55, 8.81] | 0.83 (0.49, 1.39) |  | 1.38 (0.82, 2.33) |  | 1.62 (0.96, 2.73) |  | 1.23 (0.74, 2.05) |  |
| 2nd Quartile [8.25, 8.54] | 0.99 (0.60, 1.63) |  | 1.10 (0.64, 1.89) |  | 1.39 (0.82, 2.35) |  | 0.89 (0.53, 1.51) |  |
| 1st Quartile [7.25, 8.24] | REF |  | REF |  | REF |  | REF |  |
|  |  |  |  |  |  |  |  |  |
| TG/HDL-C |  | 0.29 |  | **<0.01** |  | **<0.01** |  | **<0.001** |
| 4th Quartile [2.75, 9.30] | 1.30 (0.78, 2.14) |  | **1.73 (1.01, 2.97)** |  | **1.73 (1.02, 2.93)** |  | **1.76 (1.07, 2.90)** |  |
| 3rd Quartile [1.96, 2.74] | 1.05 (0.63, 1.74) |  | **2.21 (1.31, 3.71)** |  | **2.14 (1.29, 3.55)** |  | 1.62 (0.99, 2.68) |  |
| 2nd Quartile [1.30, 1.95] | 1.25 (0.76, 2.05) |  | **1.82 (1.07, 3.11)** |  | 1.50 (0.89, 2.52) |  | 1.03 (0.60, 1.74) |  |
| 1st Quartile [0.45, 1.29] | REF |  | REF |  | REF |  | REF |  |

**Abbreviations:** HOMA-IR, homeostatic model assessment of insulin resistance; HF, high frequency; LF, low frequency; RMSSD, root mean square of successive differences in normal-to-normal R-R intervals; SDNN, standard deviation of normal-to-normal R-R intervals; TG/HDL-C, triglyceride to high-density lipoprotein cholesterol ratio; TyG, triglyceride and glucose index. **Notes:** Estimates are adjusted for age at ancillary visit, sex, and race/study-site. A Cochran-Armitage test for trend was used to test for trend by quartiles.

**Supplemental Table 2.** Participant characteristics by quartiles of triglyceride and glucose index (TyG) (Visit 5, 2011-2013).

|  | TyG | | | | |  |
| --- | --- | --- | --- | --- | --- | --- |
|  | Quartile 1 | Quartile 2 | Quartile 3 | Quartile 4 | All | P-value |
|  | [7.25, 8.24] | [8.25, 8.54] | [8.55, 8.81] | [8.82, 9.93] |  |  |
|  | (n=187) | (n=187) | (n=187) | (n=188) | (n=749) |  |
| Age (years), mean ± SE | 78 ± 0.4 | 78 ± 0.4 | 77 ± 0.3 | 77 ± 0.3 | 78 ± 0.2 | 0.15 |
| Women, n(%) | 119 (64) | 121 (65) | 128 (68) | 120 (64) | 497 (66) | 0.78 |
| African American, n(%) | 131 (70) | 116 (62) | 103 (55) | 81 (43) | 438 (58) | <0.0001 |
| Waist circumference (cm), mean ± SE | 93 ± 0.9 | 96 ± 1 | 99 ± 1 | 101 ± 0.9 | 97 ± 0.5 | <0.0001 |
| BMI (kg/m^2^), mean ± SE | 27 ± 0.4 | 29 ± 0.4 | 29 ± 0.5 | 30 ± 0.4 | 29 ± 0.2 | <0.001 |
| SBP (mmHg), mean ± SE | 129 ± 1.4 | 127 ± 1.4 | 128 ± 1.3 | 129 ± 1.2 | 129 ± 0.7 | 0.76 |
| DBP (mmHg), mean ± SE | 64 ± 0.8 | 64 ± 0.8 | 65 ± 0.7 | 65 ± 0.7 | 65 ± 0.4 | 0.92 |
| Heart rate (beats per minute), mean ± SE | 72 ± 0.7 | 71 ± 0.7 | 73 ± 0.7 | 72 ± 0.6 | 72 ± 0.3 | 0.48 |
| Blood pressure medication, n(%) | 128 (70) | 118 (64) | 124 (68) | 131 (70) | 510 (68) | 0.51 |
| Prior CHD, n(%) | 24 (13) | 21 (11) | 25 (13) | 21 (11) | 91 (12) | 0.79 |
| Current smoker, n(%) | 8 (5) | 11 (6) | 9 (5) | 11 (6) | 39 (5) | 0.72 |
| Current drinker, n(%) | 45 (26) | 51 (29) | 66 (37) | 75 (40) | 238 (33) | <0.01 |
| Former smoker, n(%) | 71 (45) | 72 (44) | 80 (49) | 97 (56) | 325 (49) | 0.03 |
| Former drinker, n(%) | 67 (38) | 64 (37) | 56 (31) | 65 (35) | 258 (36) | 0.31 |

Participant characteristics were described overall and by quartiles of insulin resistance indexes. Continuous variables were described using ANOVA and categorical variables were described using the chi-squared test.

**Supplemental Table 3.** Participant characteristics by quartiles of triglyceride to high-density lipoprotein cholesterol ratio (TG/HDL-C) (Visit 5, 2011-2013).

|  | TG/HDL-C | | | | |  |
| --- | --- | --- | --- | --- | --- | --- |
|  | Quartile 1 | Quartile 2 | Quartile 3 | Quartile 4 | All | P-value |
|  | [0.45, 1.29] | [1.30, 1.95] | [1.96, 2.74) | [2.75, 9.30] |  |  |
|  | (n=187) | (n=188) | (n=187) | (n=188) | (n=750) |  |
| Age (years), mean ± SE | 78 ± 0.4 | 77 ± 0.3 | 78 ± 0.4 | 77 ± 0.3 | 78 ± 0.2 | 0.30 |
| Women, n(%) | 143 (77) | 117 (62) | 122 (65) | 107 (57) | 497 (66) | <0.001 |
| African American, n(%) | 127 (68) | 121 (64) | 104 (56) | 80 (43) | 438 (58) | <0.0001 |
| Waist circumference (cm), mean ± SE | 91 ± 0.9 | 97 ± 0.9 | 100 ± 0.9 | 102 ± 0.9 | 97 ± 0.5 | <0.0001 |
| BMI (kg/m^2^), mean ± SE | 27 ± 0.4 | 28 ± 0.4 | 29 ± 0.5 | 30 ± 0.4 | 29 ± 0.2 | <0.0001 |
| SBP (mmHg), mean ± SE | 131 ± 1.4 | 127 ± 1.4 | 128 ± 1.3 | 129 ± 1.3 | 129 ± 0.7 | 0.19 |
| DBP (mmHg), mean ± SE | 64 ± 0.8 | 65 ± 0.8 | 64 ± 0.7 | 66 ± 0.8 | 65 ± 0.4 | 0.51 |
| Heart rate (beats per minute), mean ± SE | 72 ± 0.7 | 72 ± 0.7 | 73 ± 0.7 | 71 ± 0.6 | 72 ± 0.3 | 0.33 |
| Blood pressure medication, n(%) | 125 (68) | 120 (65) | 130 (71) | 127 (68) | 510 (68) | 0.58 |
| Prior CHD, n(%) | 22 (12) | 20 (11) | 18 (10) | 31 (17) | 91 (12) | 0.22 |
| Current smoker, n(%) | 4 (2) | 11 (6) | 13 (7) | 11 (6) | 39 (5) | 0.11 |
| Current drinker, n(%) | 51 (29) | 64 (36) | 58 (33) | 64 (35) | 238 (33) | 0.40 |
| Former smoker, n(%) | 69 (44) | 75 (46) | 83 (51) | 93 (53) | 325 (49) | 0.06 |
| Former drinker, n(%) | 59 (34) | 61 (34) | 65 (37) | 68 (37) | 258 (36) | 0.46 |

Participant characteristics were described overall and by quartiles of insulin resistance indexes. Continuous variables were described using ANOVA and categorical variables were described using the chi-squared test.

**Supplemental Table 4.** Odds ratios and 95% confidence intervals for additional analysis, adjusting for any prior coronary heart disease

|  | Low SDNN |  | Low RMSSD |  | Low LF |  | Low HF |  |
| --- | --- | --- | --- | --- | --- | --- | --- | --- |
|  | (<97 ms) |  | (<21 ms) |  | (<10.97 Hz) |  | (<7.59 Hz) |  |
|  | OR (95% CI) | P-value | OR (95% CI) | P-value | OR (95% CI) | P-value | OR (95% CI) | P-value |
| HOMA-IR |  | 0.14 |  | **0.03** |  | **0.02** |  | **0.02** |
| 4th Quartile [4.35,16.88] | 1.31 (0.78, 2.21) |  | **1.71 (1.02, 2.87)** |  | 1.64 (0.97, 2.78) |  | **1.91 (1.14, 3.20)** |  |
| 3rd Quartile [2.79, 4.34] | 1.24 (0.74, 2.08) |  | 1.41 (0.84, 2.35) |  | **2.24 (1.34, 3.75)** |  | **1.68 (1.01, 2.81)** |  |
| 2nd Quartile [1.74, 2.78] | 0.96 (0.57, 1.64) |  | 0.95 (0.56, 1.62) |  | 1.33 (0.78, 2.25) |  | 1.20 (0.71, 2.04) |  |
| 1st Quartile [0.20, 1.73] | REF |  | REF |  | REF |  | REF |  |
|  |  |  |  |  |  |  |  |  |
| TyG |  | 0.29 |  | **<0.0001** |  | **<0.01** |  | **<0.01** |
| 4th Quartile [8.82, 9.93] | 1.35 (0.83, 2.20) |  | **2.03 (1.21, 3.39)** |  | **2.01 (1.19, 3.38)** |  | **1.99 (1.21, 3.27)** |  |
| 3rd Quartile [8.55, 8.81] | 0.82 (0.49, 1.38) |  | 1.39 (0.83, 2.33) |  | 1.62 (0.95, 2.74) |  | 1.24 (0.74, 2.06) |  |
| 2nd Quartile [8.25, 8.54] | 1.00 (0.60, 1.64) |  | 1.10 (0.64, 1.89) |  | 1.40 (0.83, 2.38) |  | 0.90 (0.53, 1.52) |  |
| 1st Quartile [7.25, 8.24] | REF |  | REF |  | REF |  | REF |  |
|  |  |  |  |  |  |  |  |  |
| TG/HDL-C |  | 0.29 |  | **<0.01** |  | **<0.01** |  | **<0.001** |
| 4th Quartile [2.75, 9.30] | 1.29 (0.78, 2.13) |  | **1.74 (1.02, 2.98)** |  | **1.72 (1.02, 2.91)** |  | **1.76 (1.07, 2.91)** |  |
| 3rd Quartile [1.96, 2.74) | 1.07 (0.64, 1.78) |  | **2.20 (1.30, 3.71)** |  | **2.19 (1.31, 3.64)** |  | 1.63 (0.99, 2.70) |  |
| 2nd Quartile [1.30, 1.95] | 1.26 (0.76, 2.07) |  | **1.82 (1.07, 3.12)** |  | 1.52 (0.90, 2.55) |  | 1.03 (0.60, 1.75) |  |
| 1st Quartile [0.45, 1.29] | REF |  | REF |  | REF |  | REF |  |

**Abbreviations:** HOMA-IR, homeostatic model assessment of insulin resistance; HF, high frequency; LF, low frequency; RMSSD, root mean square of successive differences in normal-to-normal R-R intervals; SDNN, standard deviation of normal-to-normal R-R intervals; TG/HDL-C, triglyceride to high-density lipoprotein cholesterol ratio; TyG, triglyceride and glucose index. **Notes:** Estimates are adjusted for age at ancillary visit, sex, race/study-site, and any prior coronary heart disease. A Cochran-Armitage test for trend was used to test for trend by quartiles.

**Supplemental Table 5.** Odds ratios and 95% confidence intervals for additional analysis, adjusting for systolic blood pressure

|  | Low SDNN | Low RMSSD | Low LF | Low HF |
| --- | --- | --- | --- | --- |
|  | (<97 ms) | (<21 ms) | (<10.97 Hz) | (<7.59 Hz) |
|  | OR (95% CI) | OR (95% CI) | OR (95% CI) | OR (95% CI) |
| HOMA-IR |  |  |  |  |
| 4th Quartile [4.35,16.88] | 1.27 (0.75, 2.14) | **1.72 (1.01, 2.91)** | 1.65 (0.97, 2.82) | **1.93 (1.14, 3.27)** |
| 3rd Quartile [2.79, 4.34] | 1.20 (0.72, 2.02) | 1.45 (0.86, 2.44) | **2.28 (1.35, 3.85)** | **1.70 (1.01, 2.87)** |
| 2nd Quartile [1.74, 2.78] | 0.98 (0.58, 1.66) | 0.98 (0.57, 1.68) | 1.39 (0.82, 2.38) | 1.25 (0.73, 2.13) |
| 1st Quartile [0.20, 1.73] | REF | REF | REF | REF |
|  |  |  |  |  |
| TyG |  |  |  |  |
| 4th Quartile [8.82, 9.93] | 1.31 (0.80, 2.15) | **1.96 (1.16, 3.30)** | **1.97 (1.16, 3.35)** | **1.97 (1.19, 3.26)** |
| 3rd Quartile [8.55, 8.81] | 0.81 (0.48, 1.36) | 1.30 (0.77, 2.20) | 1.55 (0.90, 2.64) | 1.18 (0.70, 1.97) |
| 2nd Quartile [8.25, 8.54] | 0.98 (0.59, 1.61) | 1.05 (0.61, 1.80) | 1.37 (0.81, 2.34) | 0.86 (0.50, 1.47) |
| 1st Quartile [7.25, 8.24] | REF | REF | REF | REF |
|  |  |  |  |  |
| TG/HDL-C |  |  |  |  |
| 4th Quartile [2.75, 9.30] | 1.26 (0.76, 2.10) | 1.70 (0.99, 2.91) | **1.71 (1.00, 2.91)** | **1.76 (1.06, 2.92)** |
| 3rd Quartile [1.96, 2.74] | 1.06 (0.63, 1.77) | **2.11 (1.25, 3.58)** | **2.16 (1.30, 3.62)** | 1.57 (0.95, 2.62) |
| 2nd Quartile [1.30, 1.95] | 1.27 (0.77, 2.11) | **1.74 (1.01, 3.00)** | 1.48 (0.88, 2.52) | 0.98 (0.57, 1.68) |
| 1st Quartile [0.45, 1.29] | REF | REF | REF | REF |

**Abbreviations:** HOMA-IR, homeostatic model assessment of insulin resistance; HF, high frequency; LF, low frequency; RMSSD, root mean square of successive differences in normal-to-normal R-R intervals; SDNN, standard deviation of normal-to-normal R-R intervals; TG/HDL-C, triglyceride to high-density lipoprotein cholesterol ratio; TyG, triglyceride and glucose index. **Notes:** Estimates are adjusted for age at ancillary visit, sex, race/study-site, any prior coronary heart disease, and systolic blood pressure.

**Supplemental Table 6.** Odds ratios and 95% confidence intervals for African American participants

|  | Low SDNN | Low RMSSD | Low LF | Low HF |
| --- | --- | --- | --- | --- |
|  | (<97 ms) | (<21 ms) | (<10.97 Hz) | (<7.59 Hz) |
|  | OR (95% CI) | OR (95% CI) | OR (95% CI) | OR (95% CI) |
| HOMA-IR |  |  |  |  |
| 4th Quartile [4.35,16.88] | 1.00 (0.52, 1.92) | 1.22 (0.63, 2.36) | 1.13 (0.57, 2.24) | 1.10 (0.56, 2.17) |
| 3rd Quartile [2.79, 4.34] | 1.73 (0.91, 3.26) | 1.29 (0.65, 2.55) | 1.89 (0.97, 3.70) | 1.34 (0.68, 2.64) |
| 2nd Quartile [1.74, 2.78] | 1.17 (0.6, 2.27) | 0.96 (0.48, 1.91) | 1.45 (0.75, 2.81) | 1.10 (0.56, 2.15) |
| 1st Quartile [0.20, 1.73] | REF | REF | REF | REF |
|  |  |  |  |  |
| TyG |  |  |  |  |
| 4th Quartile [8.82, 9.93] | 1.18 (0.64, 2.17) | **2.46 (1.3, 4.65)** | **2.15 (1.12, 4.13)** | 1.75 (0.92, 3.31) |
| 3rd Quartile [8.55, 8.81] | 0.55 (0.29, 1.04) | 1.06 (0.55, 2.06) | 1.72 (0.92, 3.23) | 0.93 (0.49, 1.77) |
| 2nd Quartile [8.25, 8.54] | 0.70 (0.39, 1.27) | 0.85 (0.44, 1.64) | 1.52 (0.82, 2.8) | 0.89 (0.48, 1.65) |
| 1st Quartile [7.25, 8.24] | REF | REF | REF | REF |
|  |  |  |  |  |
| TG/HDL-C |  |  |  |  |
| 4th Quartile [2.75, 9.30] | 1.23 (0.65, 2.3) | **2.21 (1.11, 4.39)** | **3.00 (1.55, 5.84)** | 1.90 (0.99, 3.63) |
| 3rd Quartile [1.96, 2.74] | 0.73 (0.39, 1.37) | **1.95 (1.03, 3.7)** | **2.39 (1.28, 4.45)** | 1.51 (0.82, 2.76) |
| 2nd Quartile [1.30, 1.95] | 0.87 (0.48, 1.57) | 1.05 (0.54, 2.05) | 1.43 (0.76, 2.7) | 0.70 (0.36, 1.35) |
| 1st Quartile [0.45, 1.29] | REF | REF | REF | REF |

**Abbreviations:** HOMA-IR, homeostatic model assessment of insulin resistance; HF, high frequency; LF, low frequency; RMSSD, root mean square of successive differences in normal-to-normal R-R intervals; SDNN, standard deviation of normal-to-normal R-R intervals; TG/HDL-C, triglyceride to high-density lipoprotein cholesterol ratio; TyG, triglyceride and glucose index. **Notes:** Estimates are adjusted for age at ancillary visit, sex, study-site, and any prior coronary heart disease.

**Supplemental Table 7.** Odds ratios and 95% confidence intervals for White participants

|  | Low SDNN | Low RMSSD | Low LF | Low HF |
| --- | --- | --- | --- | --- |
|  | (<97 ms) | (<21 ms) | (<10.97 Hz) | (<7.59 Hz) |
|  | OR (95% CI) | OR (95% CI) | OR (95% CI) | OR (95% CI) |
| HOMA-IR |  |  |  |  |
| 4th Quartile [4.35,16.88] | 1.89 (0.82, 4.34) | **2.47 (1.12, 5.44)** | **2.29 (1.02, 5.12)** | **2.90 (1.34, 6.27)** |
| 3rd Quartile [2.79, 4.34] | 0.96 (0.42, 2.21) | 1.54 (0.75, 3.19) | **2.58 (1.2, 5.54)** | 2.00 (0.96, 4.16) |
| 2nd Quartile [1.74, 2.78] | 0.83 (0.36, 1.92) | 0.98 (0.45, 2.09) | 1.20 (0.52, 2.75) | 1.27 (0.58, 2.77) |
| 1st Quartile [0.20, 1.73] | REF | REF | REF | REF |
|  |  |  |  |  |
| TyG |  |  |  |  |
| 4th Quartile [8.82, 9.93] | 1.77 (0.76, 4.15) | 2.02 (0.90, 4.55) | **1.83 (0.81, 4.17)** | **2.30 (1.05, 5.03)** |
| 3rd Quartile [8.55, 8.81] | 1.26 (0.50, 3.19) | 1.69 (0.74, 3.88) | **1.48 (0.62, 3.54)** | 1.59 (0.70, 3.61) |
| 2nd Quartile [8.25, 8.54] | 1.54 (0.61, 3.91) | 1.41 (0.58, 3.43) | 1.25 (0.5, 3.15) | 0.94 (0.38, 2.33) |
| 1st Quartile [7.25, 8.24] | REF | REF | REF | REF |
|  |  |  |  |  |
| TG/HDL-C |  |  |  |  |
| 4th Quartile [2.75, 9.30] | 1.62 (0.69, 3.78) | 1.77 (0.79, 3.99) | 1.17 (0.53, 2.60) | 1.87 (0.86, 4.08) |
| 3rd Quartile [1.96, 2.74] | 1.61 (0.65, 3.94) | **2.58 (1.12, 5.93)** | 1.90 (0.84, 4.27) | 1.85 (0.81, 4.22) |
| 2nd Quartile [1.30, 1.95] | 1.96 (0.79, 4.84) | **2.94 (1.24, 6.96)** | 1.54 (0.66, 3.63) | 1.48 (0.62, 3.52) |
| 1st Quartile [0.45, 1.29] | REF | REF | REF | REF |

**Abbreviations:** HOMA-IR, homeostatic model assessment of insulin resistance; HF, high frequency; LF, low frequency; RMSSD, root mean square of successive differences in normal-to-normal R-R intervals; SDNN, standard deviation of normal-to-normal R-R intervals; TG/HDL-C, triglyceride to high-density lipoprotein cholesterol ratio; TyG, triglyceride and glucose index. **Notes:** Estimates are adjusted for age at ancillary visit, sex, study-site, and any prior coronary heart disease.

**Supplemental Table 8.** Odds ratios and 95% confidence intervals for additional analysis, including other outcome metrics

|  | Low pNN50 | High LF/HF |
| --- | --- | --- |
|  | (<2.8%) | (>1.67) |
|  | OR (95% CI) | OR (95% CI) |
| HOMA-IR |  |  |
| 4th Quartile [4.35,16.88] | 1.23 (0.74, 2.05) | 1.45 (0.85, 2.47) |
| 3rd Quartile [2.79, 4.34] | 1.02 (0.61, 1.72) | 1.35 (0.78, 2.35) |
| 2nd Quartile [1.74, 2.78] | 1.35 (0.82, 2.25) | 1.32 (0.77, 2.27) |
| 1st Quartile [0.20, 1.73] | REF | REF |
|  |  |  |
| TyG |  |  |
| 4th Quartile [8.82, 9.93] | 0.90 (0.52, 1.56) | 1.12 (0.65, 1.93) |
| 3rd Quartile [8.55, 8.81] | 1.16 (0.69, 1.96) | 1.00 (0.59, 1.69) |
| 2nd Quartile [8.25, 8.54] | 1.45 (0.88, 2.38) | 1.19 (0.71, 1.99) |
| 1st Quartile [7.25, 8.24] | REF | REF |
|  |  |  |
| TG/HDL-C |  |  |
| 4th Quartile [2.75, 9.30] | 0.76 (0.45, 1.29) | 1.24 (0.73, 2.10) |
| 3rd Quartile [1.96, 2.74] | 0.91 (0.55, 1.50) | 0.97 (0.58, 1.64) |
| 2nd Quartile [1.30, 1.95] | 0.88 (0.54, 1.45) | 1.16 (0.7, 1.94) |
| 1st Quartile [0.45, 1.29] | REF | REF |

**Abbreviations:** HOMA-IR, homeostatic model assessment of insulin resistance; LF/HF, ratio of low frequency to high frequency; pNN50, the percentage of successive normal-to-normal R-R intervals that differ by >50 ms; TG/HDL-C, triglyceride to high-density lipoprotein cholesterol ratio; TyG, triglyceride and glucose index. **Notes:** Estimates are adjusted for age at ancillary visit, sex, race/study-site, any prior coronary heart disease, and systolic blood pressure.

**Supplemental Figure 1.** Study Population


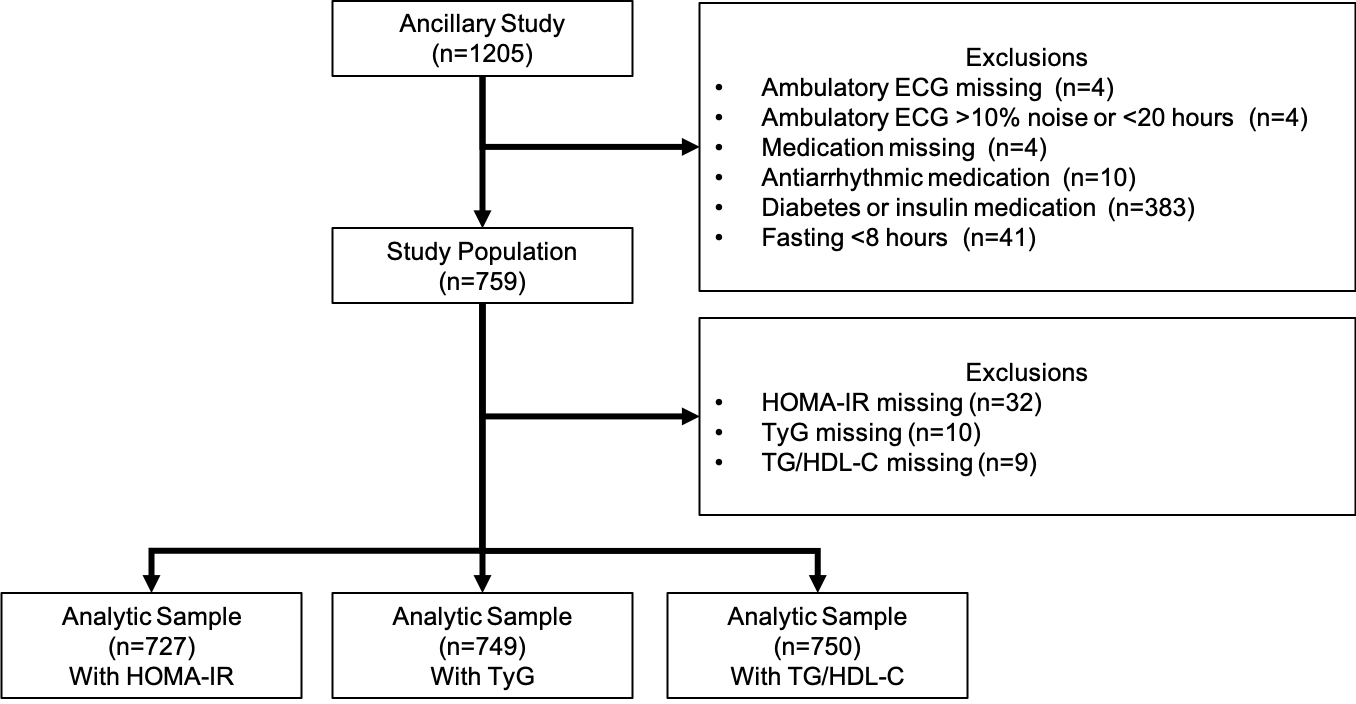

Supplement: Supplementary file 1 — Additional file 1: Table S1. Odds ratios and 95% confidence intervals for main analysis. Table S2. Participant characteristics by quartiles of triglyceride and glucose index (TyG) (Visit 5, 2011–2013). Table S3. Participant characteristics by quartiles of triglyceride to high-density lipoprotein cholesterol ratio (TG/HDL-C) (Visit 5, 2011–2013). Table S4. Odds ratios and 95% confidence intervals for additional analysis, adjusting for any prior coronary heart disease. Table S5. Odds ratios and 95% confidence intervals for additional analysis, adjusting for systolic blood pressure. Table S6. Odds ratios and 95% confidence intervals for African American participants. Table S7. Odds ratios and 95% confidence intervals for White participants. Table S8. Odds ratios and 95% confidence intervals for additional analysis, including other outcome metrics. Figure S1. Study Population. [file 12872_2020_1496_MOESM1_ESM.docx]
